# Electroanalytical overview: utilising micro- and nano-dimensional sized materials in electrochemical-based biosensing platforms

**DOI:** 10.1007/s00604-021-04913-y

**Published:** 2021-07-22

**Authors:** Robert D. Crapnell, Craig E. Banks

**Affiliations:** Faculty of Science and Engineering, Manchester Metropolitan University, Chester Street, Manchester, M1 5GD UK

**Keywords:** Biosensor, Electrochemistry, Electroanalysis, Nanomaterials, Electrochemical platforms

## Abstract

Research into electrochemical biosensors represents a significant portion of the large interdisciplinary field of biosensing. The drive to develop reliable, sensitive, and selective biosensing platforms for key environmental and medical biomarkers is ever expanding due to the current climate. This push for the detection of vital biomarkers at lower concentrations, with increased reliability, has necessitated the utilisation of micro- and nano-dimensional materials. There is a wide variety of nanomaterials available for exploration, all having unique sets of properties that help to enhance the performance of biosensors. In recent years, a large portion of research has focussed on combining these different materials to utilise the different properties in one sensor platform. This research has allowed biosensors to reach new levels of sensitivity, but we note that there is room for improvement in the reporting of this field. Numerous examples are published that report improvements in the biosensor performance through the mixing of multiple materials, but there is little discussion presented on why each nanomaterial is chosen and whether they synergise well together to warrant the inherent increase in production time and cost. Research into micro-nano materials is vital for the continued development of improved biosensing platforms, and further exploration into understanding their individual and synergistic properties will continue to push the area forward. It will continue to provide solutions for the global sensing requirements through the development of novel materials with beneficial properties, improved incorporation strategies for the materials, the combination of synergetic materials, and the reduction in cost of production of these nanomaterials.

**Graphical abstract:**

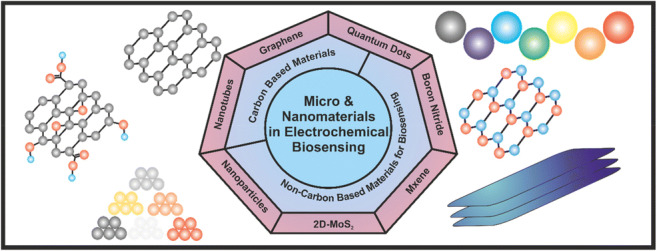

## Introduction: biosensing

Electrochemical biosensors constitute a significant portion of the huge interdisciplinary field of biosensor research. They combine the sensitivity and portability of electroanalytical methodology with the inherent selectivity of biological recognition elements [1]. In this review, we follow and focus specifically upon the IUPAC definition of electrochemical biosensors [2], and we state their exact definition, which is as follows: *An electrochemical biosensor is a self-contained integrated device, which is capable of providing specific quantitative or semi-quantitative analytical information using a biological recognition element (biochemical receptor) which is retained in direct spatial contact with an electrochemical transduction element.* These sensor platforms function through the production of an electrical signal linked to the selective reaction of the analyte and biological recognition element followed by the transduction and processing of this signal [3]. Various biological recognition elements have been utilised and reviewed such as enzymes [4] and antibodies [5], and these systems typically work through either a biocatalytic or affinity process. Biocatalytic processes, common when using enzymes, function through the production of an electroactive species upon recognition of the analyte, such as the production of hydrogen peroxide in glucose oxidase/glucose biosensors [6]. Affinity processes work through the selective binding between the analyte and recognition element, such as the interaction between a protein and antibody. The performance of developed electrochemical biosensor platforms is commonly evaluated through various experimental parameters such as the reproducibility, time of response, biosensor working lifetime, portability, linear working range, and limit of detection (LOD). The development of biosensors for healthcare applications is driven by a need to improve the current technology in terms of simplicity and speed of analysis. Northern blot methods can deliver detection limits in the nanomolar regime with excellent selectivity but is a time-consuming method; RT-PCR has very low attomolar–femtomolar detection levels and excellent selectivity but is a complex and time-consuming method; microarray methods can detect at picomolar levels but suffer from poor selectivity and is still time-consuming. Therefore, to help aid diagnosis times and reduce healthcare costs, rapid, easy-to-use biosensors with high selectivity and specificity and low LODs are required. The field of biosensors is currently progressing through the utilisation of nanomaterials, such as graphene [7–9], carbon nanotubes [10–12], and inorganic nanoparticles [13–15], explored throughout this work. The authors note that there are clear divisions among the literature on these topics between work done to progress the understanding and development of nanomaterials and work that use or combine nanomaterials for the ‘hot topic’ name recognition garnered by including them. Although the latter may be quicker and simpler, we hope researchers continue to accept the challenges presented by using new materials and the opportunities they present. These nanomaterials with unique features have helped to empower sensors to be more sensitive, precise, and reach lower LODs. The increases in sensitivity can be attributed to superior capture efficiency of the sensor, the nano-dimensions of nanomaterials comparable to the size of the target biomolecules and/or the extremely large surface-to-volume ratios of nanomaterials which enables sensors to interact with analytes much more. They provide a useful synergy between nanotechnology and the inherent advantages of electrochemical biosensing, which results in a new class of ultrasensitive and selective diagnostic tools which are low-cost, rapid, and easy-to-use [16, 17]. In this review, we will focus on some of the most common nanomaterials found in the literature, summarised in Table 1, highlighting their main characteristics, key properties, and how they have been utilised to produce effective biosensing platforms, starting with carbon nanotubes (CNTs) which have long been a staple of biosensor research.

**Table 1:** Summary of examples of different nanomaterial-based electrochemical biosensing platforms, highlighting the electrode, electrode modification, electroanalytical technique used, the analyte of interest, linear range, limit of detection, and sample medium the system was tested within

Electrode	Electrode modification	Method of detection	Analyte	Linear range	Limit of detection	Sample medium	Reference
SPE	Uricase/MWCNT	AMP	Uric acid	5–1000 μM	0.33 μM	Saliva	[18]
GC	Cyt c/TPP-HA[TFSI]/MWCNT	AMP	Hydrogen peroxide	20–892 μM	6.2 μM	Milk and juice drink	[19]
Au	PEI/CNT/Ab	Impedance	CA19–9	–	0.35 U/mL	Blood serum	[20]
GC	GDH/MG-Tb@meso	CV	Glucose	0.025–17 mM	8 μM	Blood serum	[21]
GC	phage/PEI-*f*-CNT	EIS	*Escherichia coli B*	10^3^–10^7^ CFU/mL	10^3^ CFU/mL	Culture broth	[22]
Au	AuNPs/SWCNTs/PDA	LSV	DNA	0.1 pM–10 nM	5.2 fM	Human serum	[23]
SPE	AuNP/Ab	EIS	*E. coli*	15–10^6^ CFU/mL	15 CFU/mL	PBS	[24]
Au	hCG-binding peptide	EIS	hCG	0.001–0.2 IU/mL	0.6 mIU/mL	Human serum	[25]
Ag	Au-PtNWA/PtNP/penicillinase	CV	penicillin	20–310 μM	10.5 μM	Chicken/beef extract	[26]
CCE	AuNP/antigen	LSV	TBEV	50–1600 IU/mL	50 IU/mL	Immunoglobins	[27]
GC	GOx/AgNP-MWCNT	DPV	glucose	0.025–1 mM	0.01 mM	PBS	[28]
GC	PANI/CNT/CuNP	LSV	Phthalate esters	–	0.03–0.08 nM	Bottled drinks, lake water	[29]
PG	Hb/MWCNT/CuNP/PANI	DPV	acrylamide	5–75 nM	0.2 nM	Potato crisps	[30]
GC	XO/MNP-PAMAM-PtNP/rGO-CMC	AMP	Xanthine	50 nM–12 μM	13 nM	Fish samples	[31]
ITO	Au@PtNP/GO nanozymes	AMP	Hydrogen peroxide	1–100 μM	1.62 μM	Artificial urine	[32]
GC	BNNTs-Pani-Pt-GOD	AMP	Glucose	0.01–5.5 mM	6 μM	ND	[33]
GC	BN/chitosan-Catalase	FIA	Forchlorfenuron	0.5–10.0 μM	0.07 μM	Fruits and juice samples	[34]
GC	BN–Pt NPs-GOD	AMP	Glucose	0.1–2.7 mM	14.1 μM	ND	[35]
GC	Au–Pd NPs@BNNSs/Ab_2_	DPV	*B. anthracis*	5 pg/mL to 100 ng/mL	1 pg/mL	culture broth	[36]
FTO	Apt/AuNP/BNNS	DPV	Myoglobin	0.1–100 μg/mL	34.6 ng/mL	Human serum	[37]
GC	RGO-GO_X_	AMP	Glucose	0.1–27 mM	ND	Human serum	[38]
GC	rGO-AuNR-adriamycin	DPV	Complementary DNA	1.0 × 10^−16^ to 1.0 × 10^−9^	3.5 × 10^−17^	Human serum	[39]
GC	NG-Fe_3_O_4_-MB	DPV	ssDNA	1.0 × 10^−14^ to 1.0 × 10^−6^ M	3.63 × 10^−15^ M	Human Serum	[40]
GC	3D-rGO-PANI-ssDNA-MB	DPV	breast cancer BRCA1	1.0 × 10^−15^–1.0 × 10^−7^ M)	3.01 × 10^−16^ M	Blood samples	[41]
GC	Nafion- GO_x_-G/AuNP-GC	AMP	Glucose	Low μM up to 30 mM	1 μM	ND	[42]
Pt	GO-GO_X_	AMP	Glucose	5–22 mM	ND	ND	[43]
GC	GO-AuNR-MB	DPV	Complementary DNA	1.0 × 10^−14^–1.0 × 10^−9^	3.5 × 10^−15^	ND	[44]
GC	3D GR/AuPtPd	DPV	ctDNA	0.01 to 500 pM	0.13 pM	Human serum	[45]
GC	GO-AuNR-OB	DPV	miR-155	2 fM - 8 pM	0.6 fM	Human plasma	[46]
PG	GQD/ssDNA	DPV	ssDNA or Thrombin	200–500 nM	100 nM	Buffered solution	[47]
Au	CQD/AuNP-GOx	AMP	Glucose	0.05–2.85 mM	17 μM	Human serum	[48]
CC	GOx-GQD	AMP	Glucose	5–1270 μM	1.73 μM	Human plasma	[49]
GC	DNA/chiCD	DPV	NDMA NDEA	9.9–740 nM 9.6–402 nM	9.9 nM 9.6 nM	Buffered solution	[50]
PG	CQD/ctDNA	DPV	DNR	0.1–0.5 μM	66 nM	Aqueous solution	[51]
GC	GOx/Au/MXene/Nafion	AMP	glucose	0.1–18 mM	5.9 μM	PBS	[52]
CFM	CNTs/Ti_3_C_2_T_x_/PB	AMP	Glucose lactate	10 μM–1.5 mM 0–22 mM	0.33 μM 0.67 μM	Human sweat	[53]
GC	Ti_3_C_2_-HF/TBA/GOx/GTA	AMP	glucose	50–27,750 μM	23 μM	Human serum	[54]
GC	MXene-graphene/GOx	CV	glucose	0.2–5.5 mM	0.1 mM	Human serum	[55]
GC	Ab/MXene	CV	CEA	0.0001–2000 ng/mL	0.018 pg/mL	Human serum	[56]

*PEI* polyethyleneimine, *Ab* antibody, *CNT* carbon nanotube, *PDA* polydopamine, *SWCNT* single-walled carbon nanotubes, *GDH* glucose dehydrogenase, *MG* methylene green, *Cyt c* cytochrome c, *TPP-HA[TFSI]* highly water-insoluble phosphonium-based carboxyl functionalised ionic liquid, *MWCNT* multi-walled carbon nanotube, *SPE* screen-printed electrode, *FTO* fluorine-doped tin oxide electrode, *XO* xanthine oxidase, *MNP* magnetic nanoparticles, *PAMAM* polyamidoamine G-4 dendrimers, *CMC* carboxymethylcellulose, *CCE* carbon composite electrode, *PANI* polyaniline, *PG* pencil graphite, *Hb* haemoglobin, *Apt* aptamer, *AuNP* gold nanoparticles, *BNNS* boron nitride nanosheets, *GC* glassy carbon, *GOD* glucose oxidase, *BNNTs* boron nitride nanotubes, *AMP* amperometric, *BN* boron nitride, *Pt* platinum nanoparticles, *FIA* flow injection analysis, *Ab*_*2*_ anti-*B. anthracis Sap* antibodies, *G* graphene, *AuNR* gold nanorods, *OB* anthraquinone Oracet Blue, *3D GR* 3D graphene, *ctDNA* circulating tumour DNA, *PG* pyrolytic graphite, *GQD* graphene quantum dots, *CC* carbon ceramin, *NDMA* N-nitrosodimethylamine, *NDEA* N-nitrosodiethanolamine, *DNR* daunorubicin, *TBA* tetrabutylammonium, *GTA* glutaraldehyde, *CEA* carcinoembryonic antigen

## Carbon-based materials for biosensor applications

### Carbon nanotube–based biosensors

Carbon nanotubes (CNTs) have been one of the most widely used nanomaterials in biosensor development since their re-discovery, with the name carbon nanotubes, coined in 1991 [57]; carbon fibres as small as 5 nm were originally discovered by Wiles and Abrahamson in 1978 [58]. Simply, their structure consists of tubular graphite shells. They can be described as single-walled carbon nanotubes (SWCNT) or multi-walled carbon nanotubes (MWCNT) and can vary in size from approximately 1.2–60 nm in diameter with a vast range of lengths (micron to centimetre) [12]. The variety in CNTs originates predominantly from their production method. For example, chemical vapour deposition methods produce large carbon yields and cheaper CNTs, but also generate larger defect densities that lose some of their advantageous properties [59]. It is these dimensions, along with their helical structural arrangement of carbon atoms, which introduces significant changes in the electronic density of states, providing CNTs with their unique electronic character [60]. As such, they have received an enormous amount of interest over the years due to their ultra-high specific surface area and excellent electrical conductivity and electrochemical properties [11], with significant reviews on the various applications of CNTs, such as energy applications [61–63], electronics [64–66], and biosensors [10–12, 67, 68]. To learn more about the origin of CNTs’ unique and interesting physioelectrochemical properties, we direct you to a review on the topic [69]. We will focus on highlighting key current trends in using CNTs into biosensing platforms, where they are typically functionalised with specific biorecognition elements. This functionalisation is commonly achieved through either drop-casting (taking advantage of the enhanced surface area) or covalent attachment on the tips of the CNTs, defects in the side walls and at any other non-hexagonal region [70]. Drop-casting of CNTs onto electrode surfaces, followed by drop-casting of the biorecognition element, has been commonplace throughout research in this field. A good example of this has been recently published by Shi et al. who fabricated a screen-printed electrode (SPE)–based sensor for uric acid through drop-casting of MWCNT followed by uricase onto the carbon working electrode [18]. The MWCNT were chosen for this platform due to their excellent ability to facilitate electron transfer between the analyte and electrode, in addition to the significantly enhanced surface area for uricase binding. This allowed the biosensor to achieve selective uric acid detection in the range of 5–1000 μM in only 2 min with a small sample volume of 100 μL with measurements successfully carried out in human saliva.

**Fig. 1:**
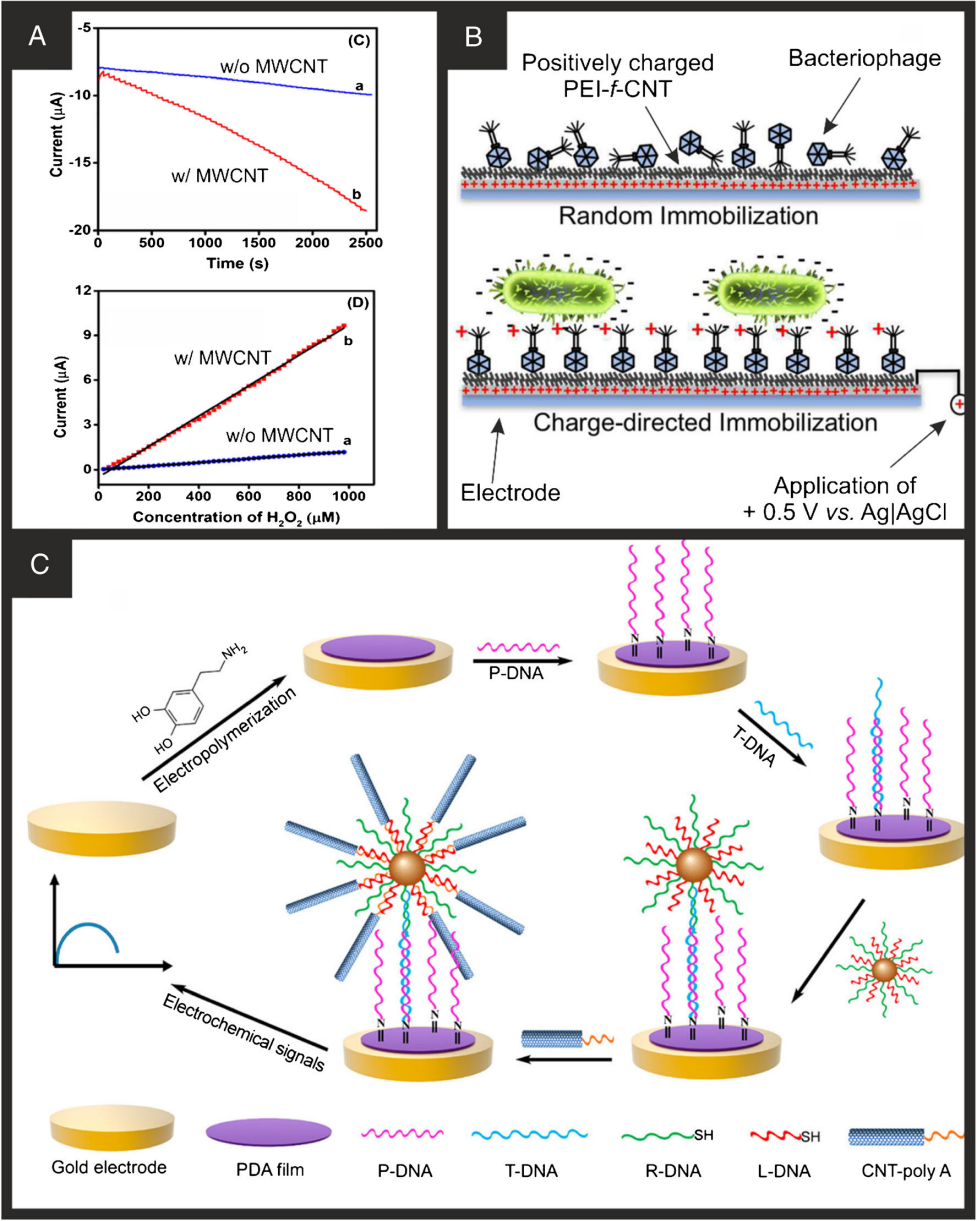
**A** (Top) Amperometric i-t curve of Cyt c/TPP-HA[TFSI]/GCE (a) and Cyt c/TPP-HA[TFSI]/MWCNT/GCE (b) upon successive additions of H_2_O_2_ into a continuously stirring nitrogen saturated phosphate buffer (0.1 M, pH = 7) with an applied potential of −0.45 V; (bottom) calibration plot for H_2_O_2_ determination. Reproduced with permission from ref. [19]. Copyright 2019 Elsevier. **B** Schematic illustration of the charge directed orientation and immobilisation of bacteriophage onto a PEI-functionalised CNT. Reproduced with permission from ref. [22]. Copyright 2020 American Chemical Society. **C** (a) Schematic illustration of the fabrication and detection process of an electrochemical DNA biosensor. Reproduced with permission from ref. [23]. Copyright 2020 American Chemical Society

Also using MWCNT drop-cast onto the electrode surface, Murphy et al. [19] produced an amperometric biosensor for the detection of hydrogen peroxide. In this system, the biorecognition element, cytochrome c, was covalently attached to a carboxylic acid functionalised ionic liquid (IL) coated onto the MWCNT/electrode surface. Both the MWCNT and IL enhanced the electron transfer of the system, contributing to a much larger amperometric response in the sensor (Fig. 1A) and producing a LOD of 6.2 μM, linear range of 20–892 μM, and a working lifetime of over 30 days. We note that care must be taken when using hydrogen peroxide in the presence of horseradish peroxidase [71, 72] as it can degrade CNTs over time. As seen, application of CNTs to the surface of electrodes for the production of biosensors is predominantly achieved through the drop-casting technique [20], and recently there has been examples reported of functionalising the CNTs before immobilisation [21]. Zhou et al. [22] demonstrate this through the functionalisation of CNTs with polyethylenimine (PEI) to produce a positively charged surface when immobilised onto the electrode (Fig. 1B). The CNTs provide excellent electron transfer properties and a large surface area for immobilisation; however, they had a measured zeta potential (PBS, pH = 7.4) of −20.2 ± 0.7 mV which would cause the opposite orientation of the phages. After functionalisation with PEI, the measured zeta potential had changed to +12.4 ± 0.8 mV. The layer of positive charge served to orientate the bacteriophage prior to its immobilisation, ensuring a uniform orientation of the biorecognition element and maximising the proportion of positively charged tail spikes available for binding to the negatively charged *E. coli*. Interestingly, the authors utilised electrochemical impedance spectroscopy (EIS) and observed a reduction in the measured charge transfer resistance (R_CT_) as more target bacteria binds to the electrode, whereas a majority of EIS-based sensors would see in increase in this parameter upon analyte binding. This is attributed to infection of the bacteria by the phage causing bacterial cell lysis and consequently the release of intracellular components to the surrounding media, possibly causing an increase in the local medium conductivity and hence a reduction in *R*_CT_ values. The sensor was able to detect the presence of *E. coli* in the range of 10^3^–10^6^ CFU/mL, with a LOD of 10^3^. Note that the unit CFU (colony forming units)/mL is a measure of the amount of viable bacterial or fungal cells per unit millilitre. Although this sensing platform has been shown to work for *E. coli*, CNTs have been shown to be degraded by certain bacteria [73] which would clearly affect possible targets and working lifetimes of sensors. As mentioned, nanomaterials have been used extensively in the development of biosensing platforms, with many reports now utilising two or more nanomaterials (nanocomposites) in conjunction to further increase the sensitivity and selectivity of the work. Recently, Han et al. [23] have shown the use of CNTs alongside Au nanoparticles (AuNPs) in ‘urchin-like’ structures for the development of a label-free biosensor for the electrochemical detection of DNA (Fig. 1C). This system takes advantage of the beneficial properties of AuNPs (discussed in more detail in the next section), especially their facile functionalisation and CNTs excellent sensitivity for monitoring chemical and environmental changes around their surfaces. In this work, a sandwich-type label-free biosensor was developed, whereby the target DNA would bind to a polydopamine (PDA) and probe DNA (p-DNA) modified Au electrode. Following this, dual-DNA (reporter and linker) modified AuNPs were introduced and bound through DNA hybridisation. Finally, end-modified CNTs were attached to the AuNPs through linker DNA, forming 3D ‘urchin-like’ nanoclusters that served to amplify the generated signal. Using linear sweep voltammetry (LSV), the sensor achieved a linear ranged of 0.1 pM–10 nM with an extremely low LOD of 5.2 fM. In this system, the flexibility of DNA strands, mass of AuNPs, and large surface area of the CNTs all work complimentary to each other, allowing the CNTs-AuNP nanoclusters to be positioned in close proximity to the electrode surface for efficient electron transport. These systems show how for the development of in vitro biosensing platforms CNT’s can provide significant benefits. However, caution must be taken when designing sensors with CNTs for in vivo biosensor platforms. Under certain conditions, nanotubes can cross membrane barriers, with suggestions that if raw materials reach organs, they can induce harmful inflammatory and fibrotic reactions [74]. In the next section, we move towards one of the most used nanomaterials in the last 15 years, graphene and its derivatives.

### Graphene, graphene oxide, and reduced graphene oxide–based biosensors

The IUPAC definition of graphene is that it is a single carbon layer of the graphite structure, describing its nature by analogy to a polycyclic aromatic hydrocarbon of quasi-infinite size [75]. Graphene has attracted a substantial interest due to its reported beneficial properties, biocompatibility, enhanced signal response, and a large surface area of 2630 m^2^/g, which is the surface area for both sides of a graphene sheet [76, 77]. It is this surface area, along with the excellent physical properties of graphene (electronic, mechanical, thermal, and optical) that has led to the extensive research in this area. There are three main forms of graphene that are commonly found in literature for use in biosensor development: pristine graphene, graphene oxide, and reduced graphene oxide. It appears that in the literature, there are examples referring to all of these as simply ‘graphene’ in their titles, which can be misleading. To form graphene on a large scale, there have been reports of both top-down and bottom-up synthesis routes. The top-down involves chemical vapour deposition of hydrocarbons onto transition metal substrates, whereas bottom-up focusses on processing pristine graphite or graphite oxide; we point you to excellent reviews on these topics [78–81]. The graphite oxide route is popular as it is exfoliated significantly more easily than its pristine counterpart to give graphene oxide sheets. These single-layer sheets have a carbon skeleton heavily decorated with oxygen functional groups and can give unique and intriguing electrochemical performance [82]. These graphene oxide sheets can theoretically be reduced through various methodologies (chemical, electrochemical, thermal, and catalytic) to form pristine graphene [83]. However, we note that virtually all reduction methodologies reported have yielded products that include oxygen species and defects in the carbon structure [84]. This can result is many different structures and properties all claiming to be graphene. As such, we implore researchers to use the term reduced graphene oxide in this case to help clarify the field.

**Fig. 2:**
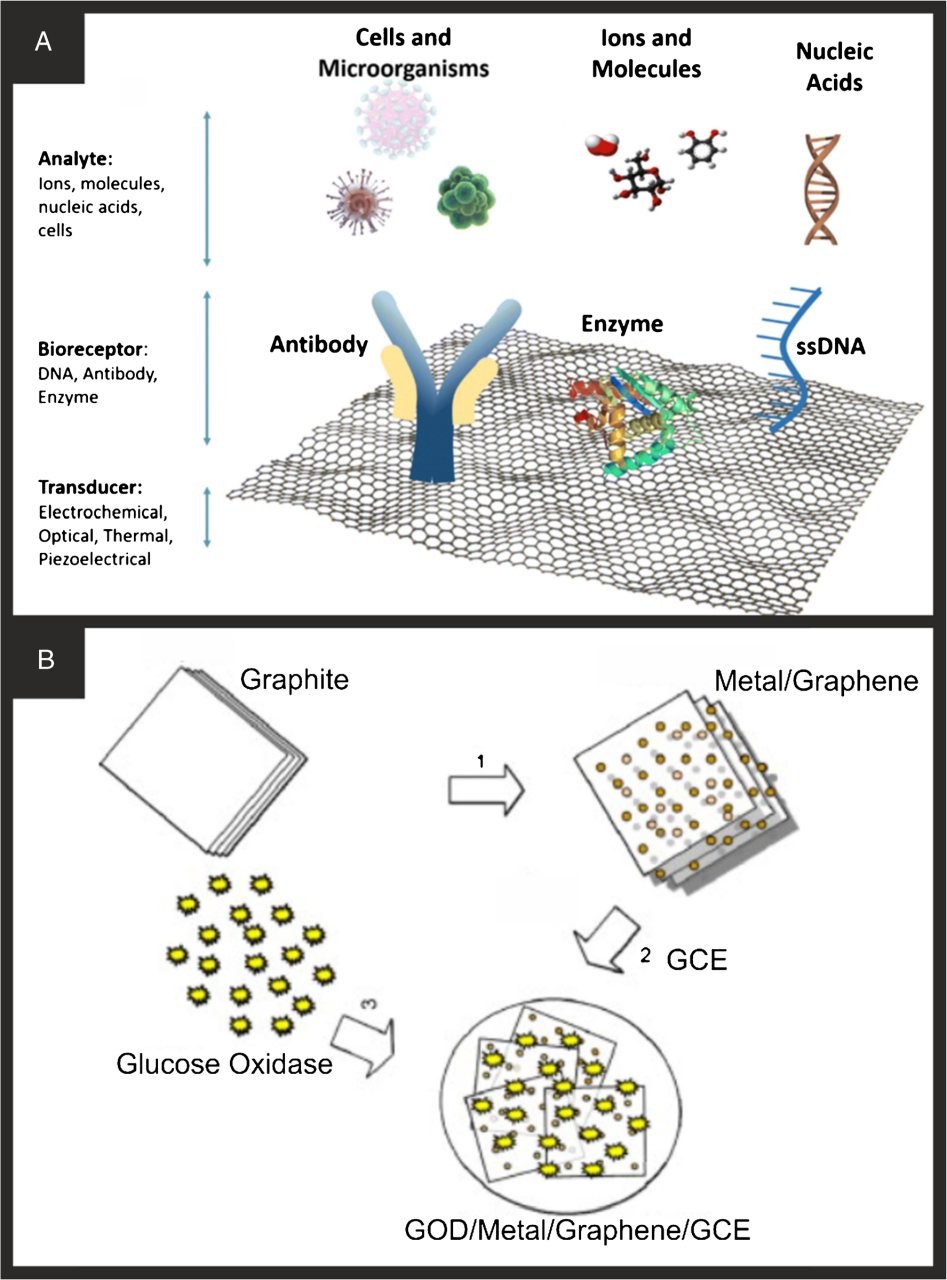
**A** Schematic illustration of examples of biosensors and components on graphene. Reproduced with permission from ref. [76]. Copyright 2018 Springer Nature. **B** Schematic of the GOD/metal/graphene/GCE bioelectrode. Reproduced with permission from ref. [42]. Copyright 2010 Elsevier

**Fig. 3:**
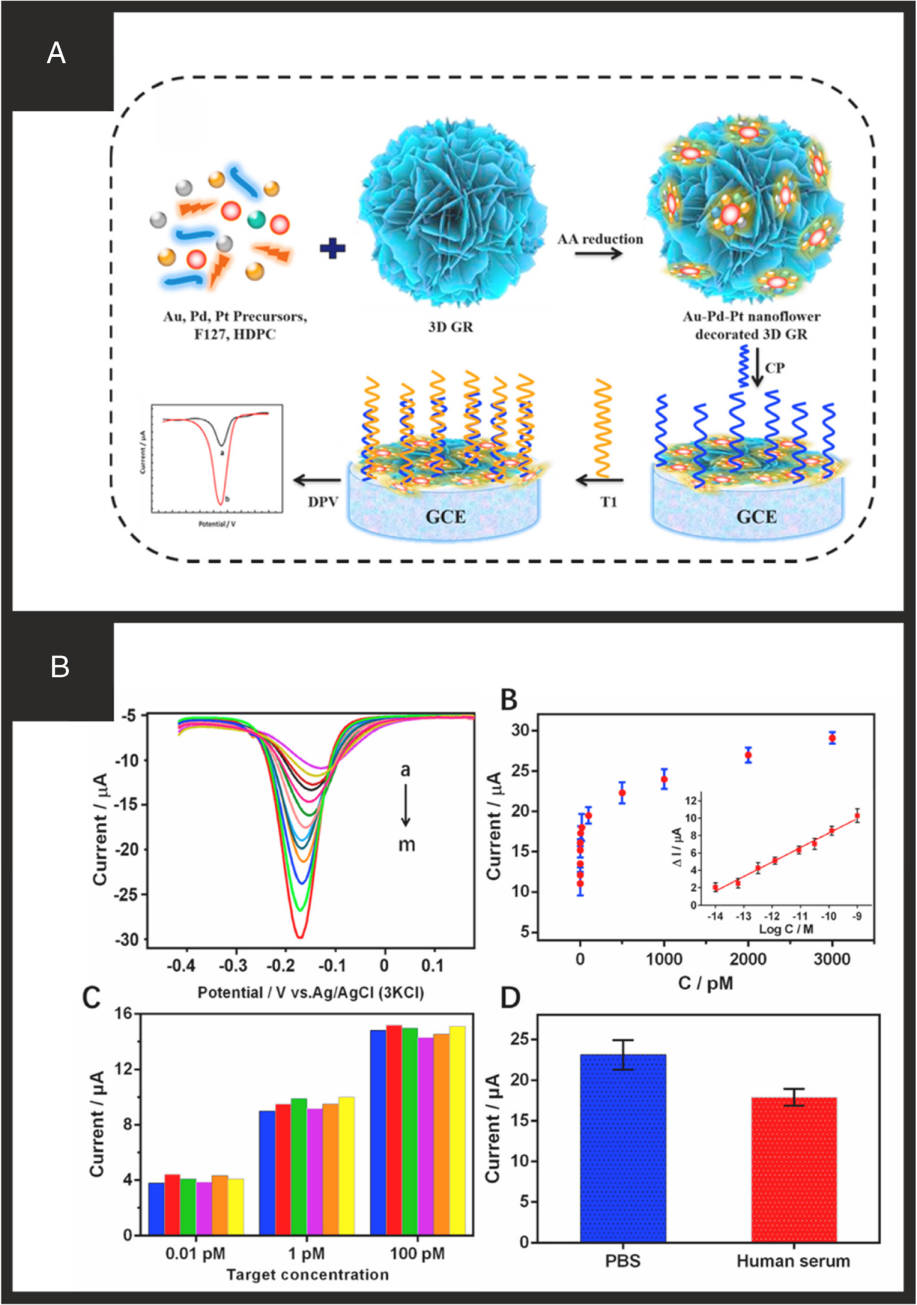
**A** Schematic for the production of the 3D GR/AuPtPd nanoflower biosensor. **B** (A) SEM images of the 3D GR nanosheets and (B) AuPtPd nanoflower structures. (C, D) TEAM and (E) high-resolution TEM images of the 3D GR/AuPtPd. (F) SEM-EDS profile and (G) EDS elemental mapping image of the 3D GR/AUPtPd. **C** Analytical performance of the DNA biosensor: (A) DPV of the system with an increasing concentration of target DNA. (B) Linear relationship between current response suppression and target concentration. (C) Reproducibility of the electrochemical biosensor in different target concentrations. (D) DPV peak current intensity for detecting *EGFR* (500 pM) in PBS and human serum. Reproduced with permission from ref. [45]. Copyright 2021 Elsevier

Figure 2A shows a schematic overview of how graphene nanobiosensors are fabricated with the graphene providing the underlying supporting electrode surface, usually immobilised upon an electrode surface (e.g. glassy carbon) onto which various sensors can be classed into using antibodies, enzymes, and ssDNA to measure cells and microorganisms, ions and molecules, and nuclei acids, respectively. The further use of nanotechnology is employed to modify the graphene surface with a chosen metallic nanoparticle which can be used to anchor the biochemical receptor. Due to the substantial interest in graphene, there are far too many papers to review and report, but we highlight current trends and approaches. One approach using pristine graphene was reported by Qi et al. [85] through drop-casting for the detection of ractopamine (RAC). This system worked through a competitive mechanism, whereby the more RAC in free solution, the less the specific antibodies would bind to the surface immobilised RAC. This system relies on full blocking of the electrode, so more RAC cannot immobilise onto the electrode as it is immobilised through π-π interactions. Additionally, there was little oxygen found in the system which could indicate some defects in the graphene structure. Building upon these studies, many approaches throw nanoparticles into the mix, as shown in Fig. 2B which can help with reducing agglomeration of the graphene sheets. Baby et al. [42] report a typical approach where graphene is produced via an exfoliation methodology, which is then decorated with the chosen metallic nanoparticles, which are then drop casted upon an electrode surface onto which GO_x_ and Nafion are added. In their approaches, they were able to detect glucose from low micromolar to high (30 mM) concentrations with a LOD of 1 μM. In such approaches, the reported benefit is justified due to the following: (1) the large surface of the graphene; (2) the large surface area of metallic nanoparticles; (3) (1) and (2) while increasing the surface area of the sensor compared to the bare/underlying electrode, which gives rise to increased sensitivity also facilitates the detection of hydrogen peroxide at lower oxidation potentials reducing the effect of any potential interferents likely to be found in real sample matrixes; (4) the Nafion® helps to reduce interferents which can be optimised as needed to overcome such interferents; (5) when graphene is prepared via an acidic solution, carboxylic acid functional groups (and others) introduced at the edges and surface of graphene likely assist in the adsorption of GO_x_ enzyme. The Nafion® layer can also help with electrode stability, i.e. reducing the likely hood of the surface modified components falling off the electrode surface during measurement. Wang et al. [39] reported a graphene-modified gold nanorod electrochemical nanobiosensor for the detection of the specific-sequence target DNA where the capture probe was immobilised on the surface of the gold nanorods with Adriamycin used as an electrochemical indicator since it could be electrostatically bonded to the anionic phosphate of DNA strands. The nanobiosensor was able to detect DNA in the range of 1.0 × 10^−16^ to 1.0 × 10^−9^ M with a detection limit of 3.5 × 10^−17^ M and was applied to determine target DNA in serum samples. Other adaptions utilise nitrogen-doped graphene decorated with Fe_3_O_4_ nanoparticles, where the former was chosen due to its reported improved electrical conductivity and the later facilitating electron transfer for the sensitive detection of DNA with excellent selectivity, fast responses, a wide linear range (1.0 × 10^−14^ to 1.0 × 10^−6^ M), and a low detection limit (3.63 × 10^−15^ M) [40]. Other approaches following a similar approach have developed electrochemical nanobiosensors as an effective tool for GM crop analysis (MIR162 detection) [23]. More recent approaches have developed 3D graphene nanoflowers (3D GR) decorated with AuPtPd nanoparticles for the determination of circulating tumour DNA (ctDNA) (Fig. 3A) [45]. These 3D graphene structures were chosen due to their low charge transfer resistance, and abundance of active sites and their coupling with metal nanomaterials have exhibited improved biosensing performance. They deployed a CRISPR/Cas9 triggered entropy-driven strand displacement reaction system onto the 3D GR/AuPtPd, due to the large surface area, and excellent electron transfer properties. The sensor using differential pulse voltammetry (DPV) (Fig. 3B) exhibited a linear range from 0.01 to 500 pM with a LOD determined to be 0.13 pM, and the clinical viability of the proposed ctDNA biosensor was investigated in human serum.

It is informative to question why use a certain nanomaterial. The current academic trends usually dictate the chosen material, i.e. graphene over CNTs since the former is, at that time, in vogue; this question can be asked anytime a new nanomaterial becomes in fashion. Interestingly, to prove this point, Dalkıran and co-workers [86] considered biosensors comprised of either MWCNTs or graphene which were both modified with Co_3_O_4_ nanoparticles and chitosan and then with galactose oxidase (GaOx) immobilised via with glutaraldehyde. Both biosensors were optimised towards the sensing of galactose with the MWCNTs-based galactose biosensor providing ~1.6-fold higher sensitivity than its graphene counterpart, with its linear working range and detection limit superior to its graphene counterpart [86]. Clearly, this provides a useful control experiment to consider when claiming advantages of the chosen carbon nanomaterial. We next look towards graphene oxide and reduced graphene oxide.

**Fig. 4:**
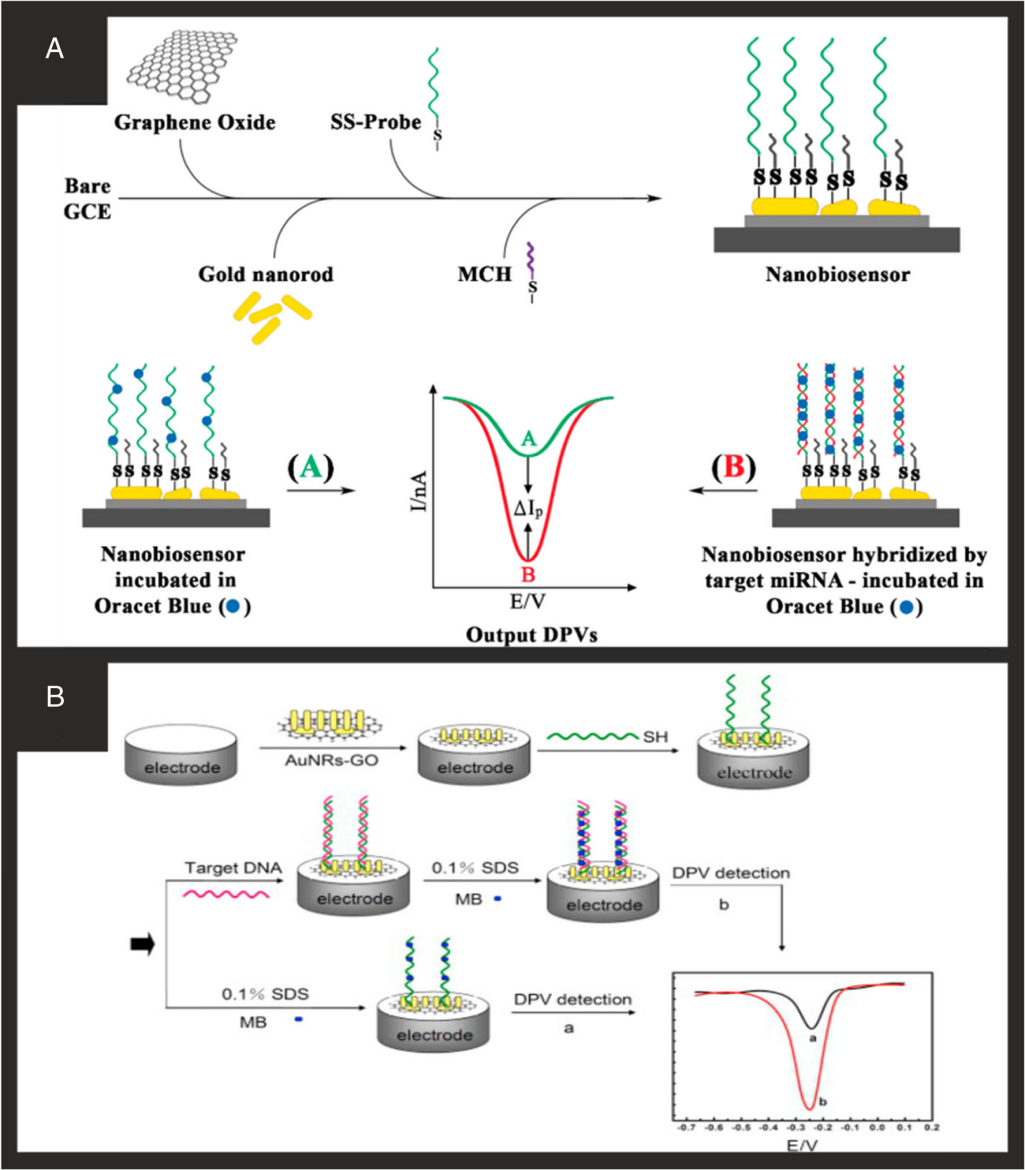
**A** A brief illustration of the assembling and working procedure of the proposed electrochemical nanobiosensor for miR-155 detection. Reproduced with permission from ref. [46]. Copyright 2016 Elsevier. **B**) Schematic representation of the DNA biosensor fabrication (MB: methylene blue; SDS: sodium dodecyl sulphate). Reproduced with permission from ref. [44]. Copyright 2013 Elsevier

Graphene oxide is well-known to be the precursor to the fabrication of graphene, but it is often overlooked and generally under-utilised as part of a composite electrode, when it has some rather interesting properties when used ‘as is’ due to it high content of oxygen functional groups for direct electroanalysis [82, 87]. One of the advantages GO has over graphene is that it can be easily dispersed in water and other polar organic solvents, and due to its unique structure, GO can be functionalised in many ways for desired applications. Reduced graphene oxide can provide a mixture of the properties from graphene and graphene oxide depending on the level of conversion from the researchers chosen reduction method. One facile approach involves the immobilisation of GOx, achieved in a single step without any cross-linking agents or modifiers [38]. GO and GO_X_ were ultrasonicated together in an aqueous solution from which an aliquot was drop casted upon a GC. This was allowed to dry at room temperature and then electrochemically reduced in a new aqueous solution to produce a GC modified with RGO-GO_X_. This biosensor exhibited a wide linear range of 0.1–27 mM to glucose and was applied to glucose determination in human serum samples under physiological conditions [38]. Utilising the benefits of the high C/O content and different moieties (e.g. carboxyl, hydroxyl, or epoxy groups), GO has been beneficially applied to the pursuit of biosensors. For example, Liu et al. [43] utilised the carboxyl groups of GO and chemically linked the amine groups of GOx through the use of 1-ethyl-3-(3-dimethylaminoprophy) carbodiimide hydrochloride (EDC) and *N*-hydroxyl succinimide (NHS). The authors used amperometry where the underlying platinum electrode was used to measure the hydrogen peroxide produced which was shown to exhibit a linear response to glucose from 5 to 22 mM with excellent reproducibility and storage stability [43]. Han and co-workers [44] reported the use of graphene oxide decorated gold nanorods fabricated with an electrostatic self-assembly technique where DPV is used to monitor the DNA hybridisation event using methylene blue as the electrochemical indicator (Fig. 4A). Under optimised conditions, complementary DNA was detected from 1.0 × 10^−9^ to 1.0 × 10^−14^ M with a detection limit of 3.5 × 10^−15^ M. While not applied in real samples, the biosensor was able to effectively distinguish complementary DNA sequences in the presence of a large amount of single-base mismatched DNA (1000:1) and demonstrated a high selectivity. GO was chosen due to its abundant oxygenated groups providing negative charges such that the sensor could be formed via electrostatic interaction with the positively charged gold nanorods which are capped with CTAB. Azimzadeh et al. [46] reported a biosensor for miR-155 detection which exhibited that wide linear range was obtained from 2 fM to 8 pM and detection limit of 0.6 fM; the various components and construction of the biosensor are shown in Fig. 4B. MiR-155 determination can provide an early detection and prognosis of breast cancer, and therefore sensitive and selective quantification in serum/plasma is required. Consequently, the authors demonstrated their biosensor in real sample analysis of human plasma at the fM level, noting that their sensor is beneficial over Northern blot which has a detection limit in nM range with excellent selectivity but is a time-consuming method. RT-PCR method has a nM–fM detection limit with excellent selectivity but is also a time-consuming and complex method. Finally, in the case of the microarray method, it has nM–pM detection limit and poor selectivity and again a time-consuming method [88]. The proposed electrochemical nanobiosensor on the other hand exhibits a femtomolar detection limit, excellent selectivity, and fast preparation and response time which suggests that it has future clinical application [46]. Again, the exact reason for the choice of graphene oxide over that say of another carbon nanomaterial and/or gold nanorods is not discussed in detail although the gold nanorods provide a surface suitable for the functionalisation procedure and the graphene oxide will provide the increased surface area for a lower cost than pristine graphene. The final carbon-based nanomaterial we will cover in this review is the use of carbon-based quantum dots, which include both carbon and graphene quantum dots.

### Carbon-based quantum dots (CQDs)–based biosensors

**Fig. 5:**
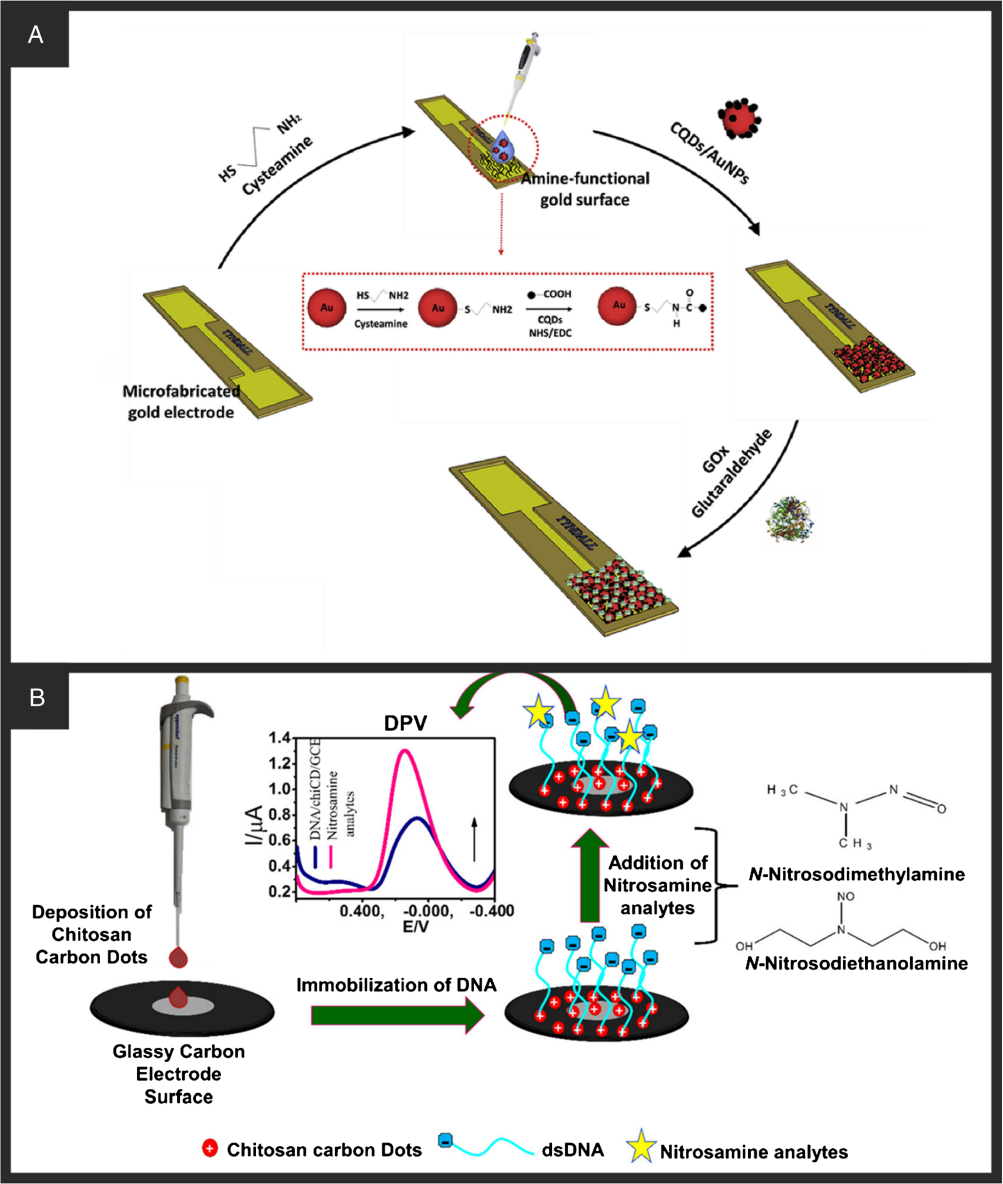
**A** Schematic illustration for the preparation of CQD/AuNP nano-hybrid materials and a schematic illustration of the immobilisation process employed in the fabrication of the biosensor. Reproduced with permission from ref. [48]. Copyright 2019 Elsevier. **B** Schematic representation of the process of fabrication the modified electrode and subsequent detection of nitrosamine. Reproduced with permission from ref. [50]. Copyright 2020 American Chemical Society

CQDs are divided into 2 subgroups, carbon quantum dots and graphene quantum dots, and can be fabricated using a range of synthesis methods with the 4 main reported to be laser ablation, microwave-assisted synthesis, electrochemical oxidation, and hydro/solvo thermal [89]. The chosen synthesis approach will result in the final structure that can range from fully amorphous through to crystalline and can result in functional groups on the surface [89]. Carbon quantum dots/graphene quantum dots generally have an average diameter of less than 10 nm and are easy to disperse in water, readily functionalised, relatively cheap, easy to prepare and non-toxic giving rise to useful properties as a component of a biosensor. As is the case with the majority of nanomaterials in biosensors, a lot of the early literature utilising quantum dots did so through drop-casting onto various substrates such as a carbon ceramic electrode for the development of a glucose biosensor [49] or onto pencil graphite [47]. Buk et al. [48] instead immobilised functionalised carbon quantum dots to prepare a glucose biosensor on an Au planar disc electrodes (Fig. 5A). The carbon quantum dots were functionalised onto AuNPs to enhance the electron transfer of the system through carbodiimide coupling chemistry. These nanohybrids were dropped onto a gold electrode followed by GOx and glutaraldehyde to immobilise the enzyme for sensing. Chronoamperometry was then used to detect glucose with a linear range of 0.05–2.85 mM and a LOD of 17 μM, with the system also successfully tested in sterile human serum samples. It is mentioned that the carboxylic acid presence on the surface of the CQDs is disrupted through the binding process to the AuNPs. This suggests the possibility of covalent functionalisation of biorecognition elements directly to the CQDs rather being drop-cast on top. Carbon quantum dots have been used in conjunction with DNA as a biorecognition element through drop-casting [51] due to their high surface area and multiple feasible binding sites. Majumdar et al. [50] reported this using carbon quantum dots from chitosan modified with DNA for the detection of mutagenic nitrosamines *N*-nitrosodimethylamine (NDMA) and *N*-nitrosodiethanolamine (NDEA). This is achieved through the drop-casting of the chitosan carbon dots onto a GCE followed by drop-casting of DNA (Fig. 5B). This produced a sensor capable of detecting both compounds with low LODs of 9.9 and 9.6 nM, respectively, through the modification of the immobilised DNA by the mutagenic *N*-nitrosamine. This system although reporting good detection capabilities has serious drawbacks as they report, through DPV measurements, that the sensor is only stable for 3 h post-production significantly hindering its real-world application. Research into carbon/graphene quantum dots has been predominantly focussed on the fluorescent properties and enhancing their capabilities for optical-based sensing. However, these examples provide evidence that there is a place for them in electrochemical biosensor research due to their beneficial properties. Although many of the systems discussed do not fully utilise the beneficial functional groups available on the surface of the quantum dots, we suggest this as an avenue for further work. We next move onto work using nanomaterials that is not solely based on carbon, starting with a continuation of this section in non-carbonaceous quantum dots.

### Nanostructure-based biosensors

Quantum dots are in effect semi-conductor nanoparticles; however, we will now move to look at metallic nanoparticles. These nanoparticles based on metals such as gold, silver, platinum, copper, and iron are typically what is meant in the literature when the simple term nanoparticle is used and offers different properties and opportunities when used in electrochemical biosensing platforms.

### Nanoparticle-based biosensors

Engineered nanoparticles can be defined both through their size (1–100 nm) and their properties (different from particles of the same composition that are not on the nanoscale) [90]. The size and morphology of nanoparticles have strong influence on their electronic, magnetic, and catalytic properties [14]. As such, they have received strong interest in a plethora of fields of research, such as medical imaging and drug delivery applications [91–93], fuel cells [94, 95], optical sensing [96, 97] and of course electrochemical sensing applications [98, 99]. Nanoparticles have been used extensively in the development of electrochemical biosensors due to their advantageous properties that they bring to the platforms. These include their greater surface area, enhanced electron transfer, plethora of functionalisation opportunities, and their ability to make their electrochemical interfaces behave as nanoelectrode ensembles, giving a larger ratio between Faradaic and capacitive currents which can lead to improvements in the LOD [14]. The authors note there is an extensive range of possible nanoparticles with wide-ranging synthesis methods, advantageous properties, and possible applications and as such; there is a plethora of review articles focussing on these that we direct you to [13, 15, 37, 100–105]. Due to this substantial interest, we will focus on highlighting key new trends and approaches in how these nanomaterials are used in current literature for the development of electrochemical biosensors.

**Fig. 6:**
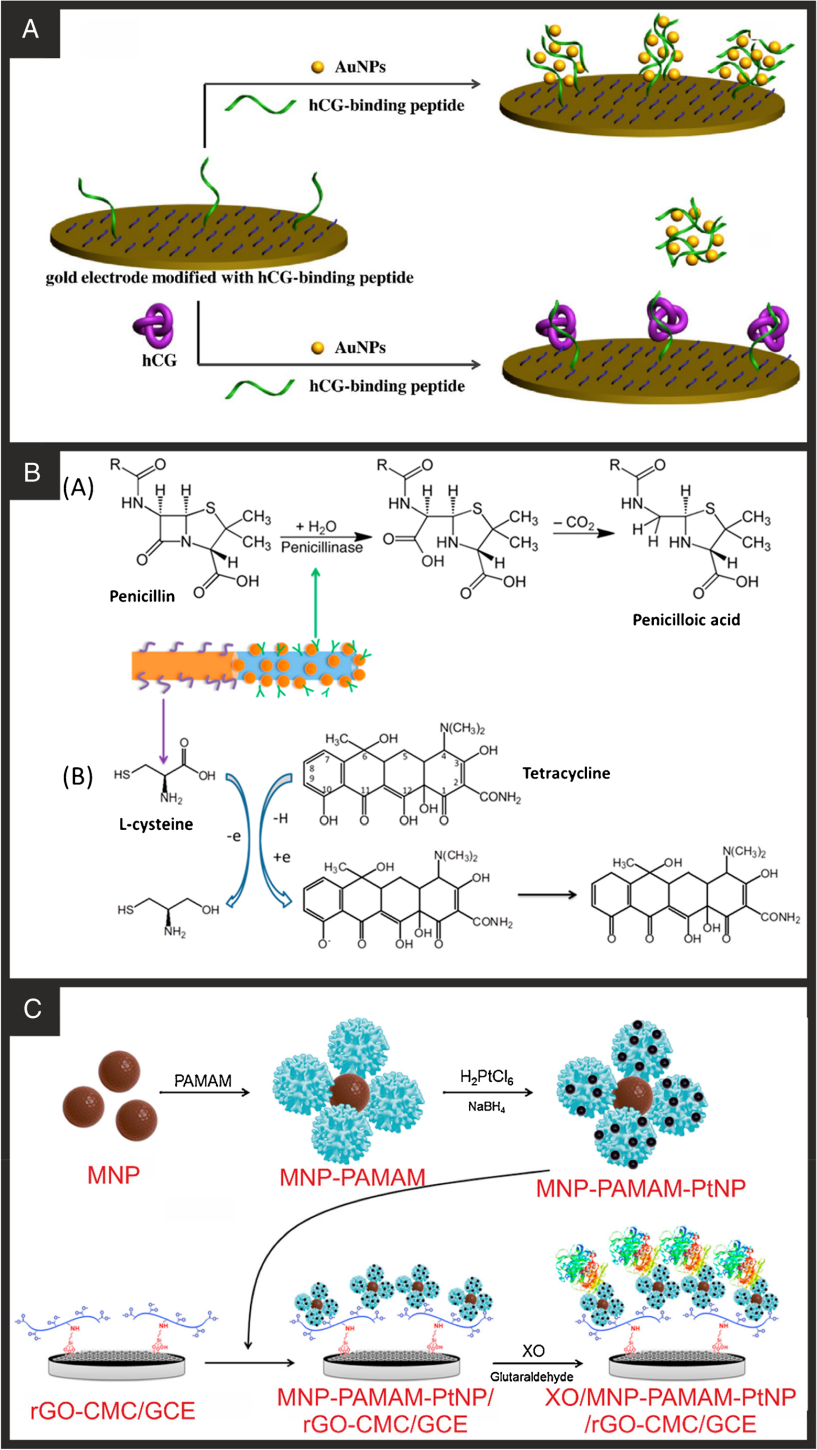
**A** Schematic illustration of the electrochemical method for hCG detection using a peptide probe as the receptor of hCG and the inducer of AuNPs assembly. Reproduced with permission from ref. [25]. Copyright 2017 Elsevier. **B** The sensing mechanism of (A) penicillinase with penicillin; (B) L-cysteine with tetracycline with Au-Pt multisegment nanowire array. Reproduced with permission from ref. [26]. Copyright 2019 Elsevier. **C** Schematic display of the preparation of the MNP-PAMAM-PtNP and the XO/MNP-PAMAM-PtNP/rGO-CMC/GCE enzyme electrode. Reproduced with permission from ref. [31]. Copyright 2016 Elsevier

Gold nanoparticles are the most commonly used nanoparticle in the development of biosensors due to their ease of preparation and biocompatibility. Although with the recent increase in cost of gold, approximately doubling in the last 10 (Cooksongold, Birmingham, UK), they are becoming a more expensive option. They also offer facile conjugation to biological recognition elements through the exploitation of the strong affinity between mercapto and amino functionalities and gold [13]. A good example of this was recently published by Vu et al. [24] in the development of an electrochemical biosensor for detecting bacterial pathogens. The authors used a facile electrochemical methodology [106] to deposit AuNPs onto the surface of SPEs, which allowed control of the size to 18.0 ± 0.6 nm and was followed by immobilisation of specific antibodies for *E. coli* O157. This was achieved through a 3-mercaptopropyl trimethoxysilane (MTS) and N-(γ-maleinidobutyryloxy) succinimide (GMBS) linkage only possible due to the presence of AuNPs on the surface of the SPE. Using EIS, this simple electrochemical platform succeeded in detecting *E. coli* O157 in the range of 10–10^6^ CFU/mL with a very low LOD of 15 CFU/mL. Typically, AuNPs would be used in this way to help increase the surface area of the electrode, aid in electron transfer, and allow for facile coupling of biological reagents. The improved electron transfer performance of the electrode is key here, as simply using the AuNPs as a linking group is not cost-effective; facile binding of biorecognition elements has been shown directly to the surface of SPEs [107]. The nanoparticles do not always need to be deposited onto the electrode surface though Han et al. [108] utilised their binding ability from solution to the sensor after attachment of the target to produce a successful electrochemical biosensor for the detection of DNA. Although this sensor showed impressive LODs and good signal response, a significant response was still observed from single and double mismatched DNA, which could lead to issues in quantitative analysis. Although this could be attributed to the biorecognition element rather than the nanomaterials themselves. Another example is presented by Xia et al. [25] who used AuNPs in free solution to help in the detection of human chorionic gonadotropin (hCG) and amyloid-β oligomer (AβO). In this report, thiolated peptides were immobilised onto a cleaned Au surface, with the remaining free surface blocked by the addition of 6-mercapto-1-hexanol (MCH) so that no non-specific binding could occur. This system could then be incubated with the sample to allow any binding between the peptide and the target before being further incubated in a free solution of AuNPs and another thiolated peptide. Sensor platforms designed like this not only utilise the improvement in electrochemical properties given by the AuNPs, but also utilise chemistry specific to them, meaning they are clearly preferred to other nanomaterials for this application. The sensor works through EIS, whereby if the immobilised peptide is free and binds to the AuNPs in solution, this can further recruit thiolated peptides and hence more AuNPs, significantly reducing the *R*_CT_ of the system (Fig. 6A). If the target is bound to the immobilised peptide, this recruitment process can no longer occur, and the *R*_CT_ remains high. In this way, hCG was able to be detected in a linear range of 0.001–0.2 IU/mL with a LOD of 0.6 mIU/mL. The authors note that IU (international unit)/mL refers to the international arbitrary amount of a substance agreed upon by scientists and doctors; in the case of hCG, a reading of above 25 mIU/mL is considered a positive result for pregnancy [109], and a result <5 mIU/mL generally indicates a negative test [110]. Another interesting use of AuNPs was reported by Li et al. [26] who created a hybrid array biosensor for the detection of penicillin and tetracycline using penicillinase and L-cysteine as biorecognition elements, respectively (Fig. 6B). To separate the two sensing areas, they formed a multisegment nanowire on the surface of a silver-coated anodic aluminium oxide membrane, first forming an Au nanowire (~2 μm) through electrodeposition at a current density of 1 mA/cm^2^ for 2 h, followed by a Pt nanowire (~2 μm) on top of the Au through electrodeposition at 1.5 mA/cm^2^ for 2 h. The electroplating solutions were thoroughly removed with deionised water after each step to ensure the precise formation of each nanowire segment. The Au nanowire segment could then be directly immobilised with a monolayer of L-cysteine to form the sensing component for the tetracycline. The Pt nanowire would not form a monolayer of L-cysteine when incubated, so could then be modified with AuNPs, using an electroless deposition [111], allowing the deposition of the penicillinase enzyme through a thiotic acid and EDC/NHS linkage. Using this methodology, the authors managed to detect both analytes successfully from 20 to 310 μm with LODs of 10.5 and 15.2 μM for penicillin and tetracycline, respectively. Again, not only the beneficial electrochemical properties and increased surface area are used, but the chemistry of the metals. This gives an elegant example of how sensor platforms can be uniquely produced by manipulating different material properties.

Other types of nanoparticles have been utilised more sparingly throughout literature for the development of electrochemical biosensors such as AgNPs [27, 28], CuNPs [29, 30], FeNPs [112, 113], and PtNPs [31, 32]. The choice between these nanoparticles typically comes down to the specific system being used, whether enhanced electron transfer, facile bio-conjugation, stability, biocompatibility, cost, or a mixture of these properties is most important. Silver nanoparticles (AgNP) are a much cheaper precious metal option than gold, with the price of silver at the time of writing ~ £19.07/Oz (compared to ~ £1292.37/Oz for gold, values obtained from Cooksongold, Birmingham, UK). Silver possesses the highest electrical and thermal conductivity and lowest contact resistance of any metal, which is why it has been used extensively in commercial manufacturing for electronics and electrochemistry. Silver nanoparticles are chemically inactive and stable in water and do not oxidise in air. They have also been shown to inhibit the growth of bacteria and other microorganisms [114], but there has also been studies indicating that AgNPs are toxic to mammalian cells, with in vivo studies suggesting that they cause toxicity in several organs (including the lungs, liver, and the brain) in rats and mice [115]. Copper nanoparticles are of interest due to their low-cost, Earth abundance and their wide range of possible oxidation states enabling both one and two electron pathways. Additionally, due to the high melting point, they could provide additional benefits for sensing in high temperature or pressure settings. Issues arise with CuNPs as they are easily oxidised leading to issues with their fabrication and stability. They are especially suited to the detection of carbohydrates and amino acids under highly alkaline conditions as they do not suffer from the electrode poisoning that Au and Pt do in these conditions [105]. As with silver, CuNPs have been shown preliminarily to cause potential toxicity in both human and ecological systems [116], especially in the liver and kidneys. Iron is another low-cost, Earth abundant and easily oxidised metallic nanopartile. This is why for electrochemical biosensing applications, research focusses on the use of iron oxide nanoparticles. It is important to note when using NPs made from metals with a passivating layer externally, it can exhibit properties which combine both the core and shell, leading to improvements in some sensing performances but can also lead to different mechanisms depending on the ratio between core and shell [117]. Platinum historically is the most expensive out of the metals discussed in this section; however the price of platinum is currently fairly similar, if not lower, to that of gold. The metal is unreactive with water, acids, or bases and does not oxidise in air. However, it can be corroded by halogens, cyanides, sulphur, and alkalis, which must be taken into account when used [105].

Borisova et al. [31] utilised PtNPs in hybrid nanomaterial layer-by-layer electrochemical biosensor structure (Fig. 6C). This involved a base of polydopamine (PDA) modified magnetic nanoparticles covalently attached to polyamidoamine G-4 dendrimers, which were then further decorated with the PtNPs. This system was used as a scaffold for the covalent attachment of xanthine oxidase through glutaraldehyde-based cross-linking and used to detect the presence of xanthine in the range of 50–1200 nM, with a LOD of 13 nM. In this system, the PtNPs introduced a larger background current but also significant electrocatalytic effects of the electrochemical oxidation of xanthine by reducing the required overpotential. This system used a modified reduced graphene oxide (rGO) base layer; this combination of synergising nanomaterials is becoming much more common in literature as published work moves from hot topic to hot topic, combining each of them. We note that it is important that work fully explains the benefit that each nanomaterial brings to every biosensor platform; otherwise the additions become redundant and would hinder the commercialisation of any real product.

More examples of nanomaterials in the electrochemical biosensor developers arsenal are 2D nanomaterials, of which graphene has already been discussed. However, there are other significant analogues of graphene that possess different properties and are more suited for certain applications which we will discuss now.

### Non-graphene-based 2D materials

The following sections focus on the use of two-dimensional (2D) materials commonly found in literature other than graphene and graphene-based derivatives. With the explosion of research into the applications of graphene and its derivatives following its discovery, these materials have significantly less research published exploring their uses in biosensor applications. There are even books focussed on the subject of 2D materials except graphene, such as the density of research in the area [118]. As such, there are review papers that focus specifically on the topic of non-graphene-based 2D materials for sensing [119, 120]. We begin alphabetically with boron nitride based biosensor developments.

### 2D hexagonal boron nitride (2D-hBN) and related boron nitride nanostructure biosensors

**Fig. 7:**
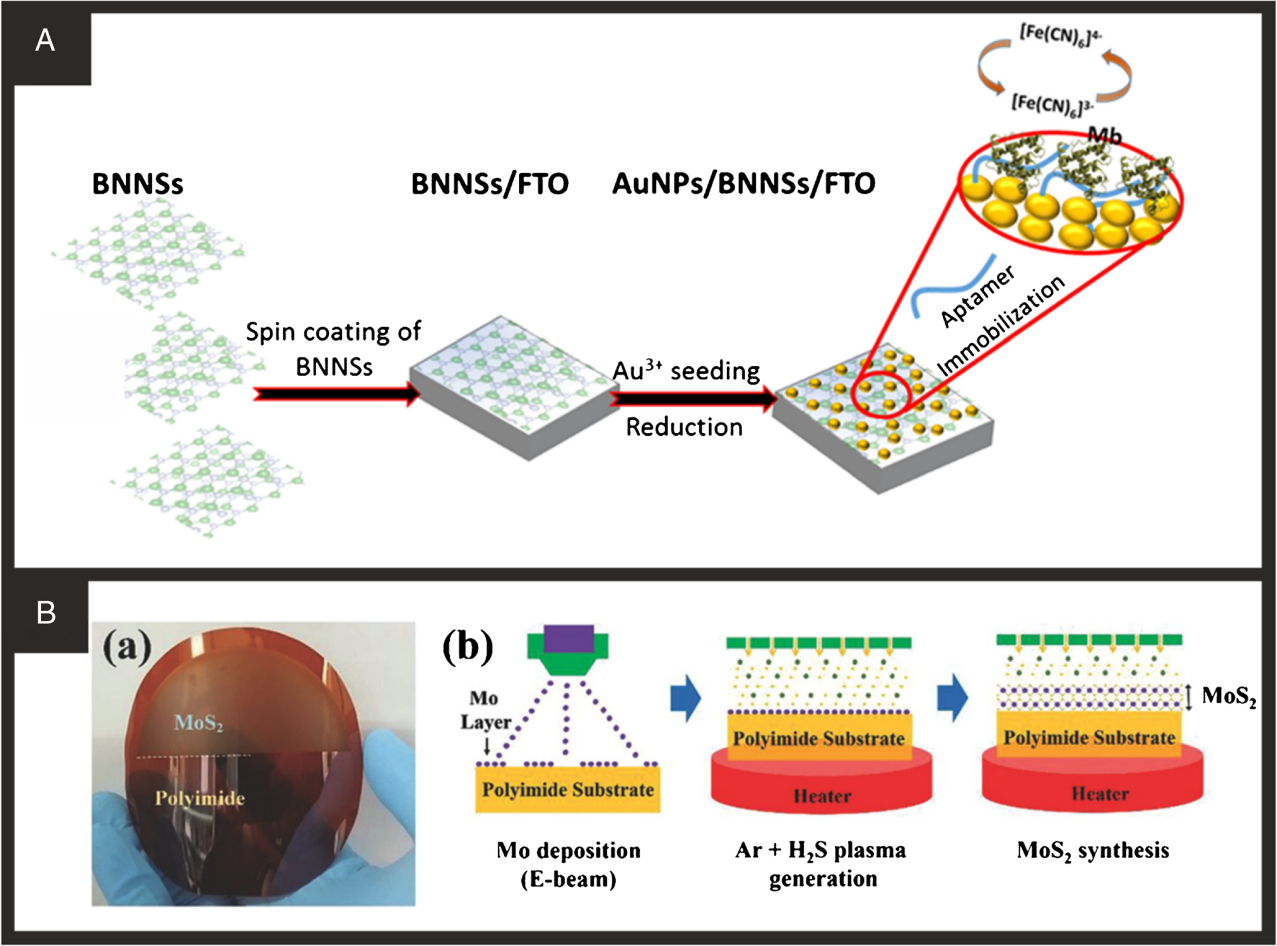
**A** Schematic illustration for the fabrication processes of the BNNS aptasensor for the detection of Mb. Reproduced with permission from ref. [37]. Copyright 2019 Elsevier. **B** Schematic illustration for the fabrication processes of the BNNS aptasensor for the detection of Mb. Reproduced with permission from ref. [124]. Copyright 2015 Wiley

2D hexagonal boron nitride (2D-hBN) is a structural analogue of graphite that presents an sp^2^ hybridisation of B–N bonds in a layered honeycomb structure comprising rings of borazine (B_3_N_3_H_6_) which are typically comprised of lateral sizes from a few hundred nanometres to tens of microns, depending on the fabrication approach employed [121–123]. BN can also exist in other structural forms such as nanotubes, fullerenes, whiskers, and quantum dots. The uptake of boron nitride into biosensing application and that of electroanalysis is limited, but through careful design and introducing defects upon its basal surface, to improve its electrochemical performance, it is slowly being explored [123]. Table 1 summarises approaches in the utilisation of BN in biosensors which have used BN nanotubes [33], naniowhiskers [35], and nanosheets [34, 36, 37]. Adeel and co-workers [37] have utilised 2D-hBN nanosheets as the basis of an aptasensor for the detection of myoglobin, a cardiac biomarker. Figure 7A overviews the construction of the aptasensor where boron nitride is first exfoliated and then spin coated onto a fluorine-doped tin oxide electrode. Next this surface is then modified with gold nanoparticles (10 nm diameter) via covalent attachment upon which a thiol-functionalised DNA aptamer via the covalent interaction of Au–S was immobilised. The authors noted that 2D-hBN was chosen over than of graphene due to it being easier to functionalise with gold nanoparticles which then acted as a specific linker with the aptamer [37]; note that this is a common theme for BN when utilised as the basis of a biosensor. The design of the aptasensor therefore allows the specific binding of the target analyte, myoglobin. In this approach, the [Fe(CN)_6_]^3−/4-^ redox probe is used to monitor the binding of the myoglobin; in the absence of any myoglobin, a large electrochemical signal is observed from the redox probe at the gold nanoparticle modified 2D-hBN, where binding blocks the electrode surface and the redox probe is unable to measured which gives rise to a ‘signal off’ type biosensor. The aptasensor exhibited a linear range from 0.1 to 100 μg/mL with a limit of detection of 34.6 ng/mL. The aptasensor was successfully applied for myoglobin sensing in human serum.

### 2D-MoS_2_ and analogous nanomaterial-based biosensors

Two-dimensional molybdenum disulphide (2D-MoS_2_) is another 2D material, like graphene and 2D-hBN previously, often seen in the literature that has recently received considerable attention in terms of energy storage [125–127], the hydrogen evolution reaction (HER) [128–130], and oxygen reduction reaction [131], among others. This material is a member of the family of 2D materials known as the transition metal dichalcogenides, which provide promising alternatives to the use of graphene. For more information on this family of materials, we direct you towards the review by Manzeli et al. [132]. Due to the robustness of MoS_2_, it has been the most studied of these materials, and therefore this section will focus predominantly on biosensors utilising that. The 2D-MoS_2_ is commonly formed when hexagonal molybdenum sulphide is exfoliated into one layer to form layers of molybdenum atoms sandwiched between sulphur atoms. These planes can be grown with large lateral dimensions with basal plane ends, which facilitates their stability in liquids and oxygen containing solutions [133]. Similarly to graphene and other 2D materials, 2D-MoS_2_ offers a large surface area which can contribute to its biosensing performance; however, it is its suitable band gap that makes it stand out when it comes to biosensing. In comparison, graphene and graphene oxides have no band gap, whereas a lot of stoichiometrically similar 2D oxides have much larger band gas requiring high energy applications. Additionally, it has been shown that both MoS_2_ and WS_2_ show very low cytotoxicity and genotoxicity highlighting that these materials may be more beneficial for the development of in vitro biosensors [134]. For more information on 2D-MoS_2_ and a deep dive on all forms of sensors made, we point the reader to the review by Kalantar-zadeh and Ou [133]. Other reviews on the use of 2D-MoS_2_ for biomedical applications [135] therapeutics, bioimaging, and biosensors [136–138] are available.

One of the first reports for the utilisation of 2D-MoS_2_ in a biosensing platform was for the detection of glucose [139]. Through simple drop-casting onto an APTES functionalised GCE it was seen that the reduction of 2D-MoS_2_ in 0.5 M NaCl led to a large improvement in the electrochemical response. This was used as a glucose biosensor through drop-casting GOx onto the surface with chitosan. The chitosan is key to this design for enhancing the immobilisation of GOx as it adheres well to the negatively charge surface of 2D-MoS_2_. This biosensing platform gave a moderate response for the detection of glucose between 0 and 20 mM but highlights how a simple method of incorporation can be improved through the addition of specific additives. There have been many examples of drop-casting of MoS_2_, similar to most of the nanomaterials covered here, in the development of biosensors for glucose [140], lactate [141], and hydrogen peroxide [142], among others. There have been reports of combining drop-cast 2D-MoS_2_ with other nanomaterials, mainly metallic nanoparticles, such as gold [143]. The majority of this work claims to be combining the beneficial properties of both nanomaterials but only quote the increased surface area of 2D-MoS_2_. Insights into the unique synergistic combination of nanomaterials is extremely important and worthwhile research; however, if increases in the surface area are the only goal, then the cheapest material possible should be utilised to produce the most cost-effective sensing platform. One example, using drop-casting, of a comparison between the biosensing performance of molybdenum and tungsten dichalcogenides is given by Rohaizad et al. [144], who show that the tungsten derivatives show faster heterogeneous electron transfer rates than their molybdenum-based counterparts when incorporated into second-generation glucose biosensors. The authors attribute this improvement to the dominance of the 1 T phase of the tungsten biosensors produced through the lithium intercalation method. This highlights the importance of preparation and characterisation of the nanomaterials used in biosensors as this 1 T phase shows metallic conductivity compared to the semiconducting 2H phase.

All of the methods presented involve a transfer step; that is, the 2D-MoS_2_ is produced separately and then incorporated onto the biosensing platform. The integration of these materials onto different substrates for sensor development can lead to defects in the material. Therefore direct synthesis of the nanomaterial onto substrates could be advantageous [124]. Kim et al. achieved this through the direct formation of 2D-MoS_2_ onto an Au electrode coated onto a flexible polyimide (PI) substrate (Fig. 7B). Mo was coated onto the Au surface at a constant rate of 0.1 Å/min to give a layer size of 1 nm. Following cleaning of the surface and equilibrium of the temperature at 150 °C, the film was sulphurised under H_2_S and Ar plasma for 1.5 h to directly form the 2D-MoS_2_ film on the surface. Note that when forming 2D materials on a surface, it is key to maintain the temperature well below the melting point of any substrate. Detection of important endocrinopathy hormones was achieved by this platform through a competitive assay procedure, whereby modified antibodies in the solution can either bind to free target in solution or immobilised targets on the electrode. The more free target present in the sample, the less antibodies will be free to bind to the immobilised target on the electrode surface causing a reduction in current response. This sensor platform was tested in serum and produced excellent results for the detection of parathyroid hormone versus pre-measured samples using a standard commercial immunoassay (E 170, Roche Diagnostics, Germany) but showed significantly worse performance for triiodothyronine and thyroxine. This system shows the benefits of being able to form the nanomaterial directly on the surface of the substrate. The next material discussed is less developed in terms of the research towards it. However, the amount of publications is rapidly growing, indicating it could be the next in the line of ‘hot topics’ in nanomaterial research.

### MXene-based biosensors

**Fig. 8:**
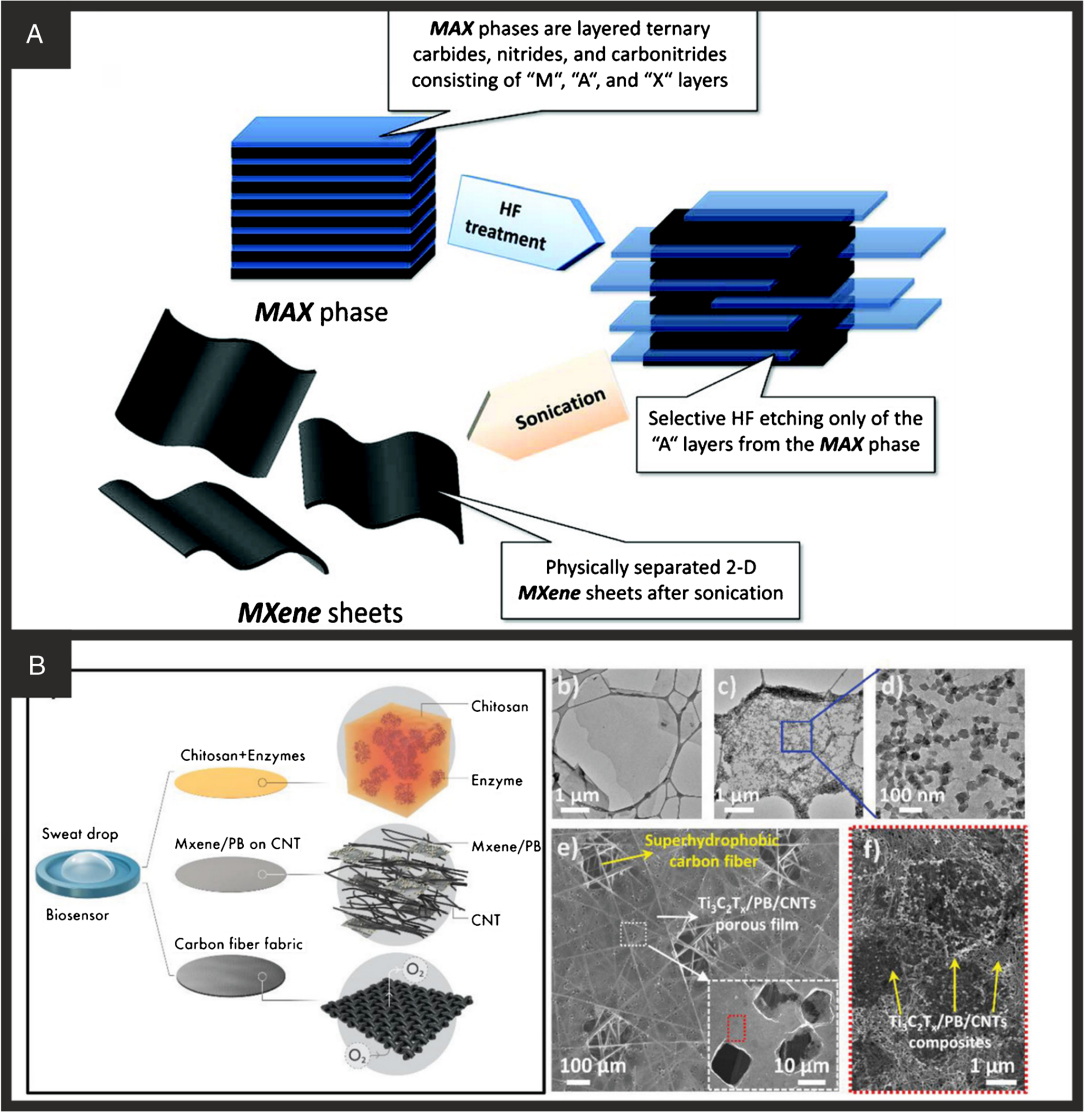
**A** Schematic for the exfoliation process of MAX phases and formation of MXenes. Reproduced with permission from ref. [145]. Copyright 2012 American Chemical Society. **B** (a) Schematic illustration of the oxygen-rich enzyme electrode, (b) TEM images of Ti_3_C_2_T_x_ nanosheets, (c,d) TEM images of the Ti_3_C_2_T_x_**/**PB composite, (e) SEM image of porous and ultrathin Ti_3_C_2_T_x_/PB and CNTs ternary film, with the inset (white box) displaying a zoomed in SEM image of the holes in the film, and (f) magnified image of the region marked by a red frame in (e), which depicts the porous and ultrathin Ti_3_C_2_T_x_/PB and CNTs ternary film. Reproduced with permission from ref. [53]. Copyright 2019 Wiley

MXenes are two-dimensional materials composed of early transitional metal carbides and carbonitrides. They are formed through the etching out of the A layer from MAX phases (Fig. 8A), whereby M is an early transition metal, A is defined as an A-group element (which mostly consist of group 13 and 14), and X is carbon and/or nitrogen [146]. For more information on the synthesis, characterisation, and alternative uses of MXenes, we direct you to some excellent review articles focussed specifically on them [145, 147, 148]. The surge of interest in researching MXenes is primarily due to their attractive properties such as high surface area, layered morphology, hydrophilicity, and high electrical conductivities. These advantageous properties have led to research using MXenes in various applications such as energy storage [149, 150], hydrogen evolution [151], and electronics [152]. Although these properties are extremely exciting, MXene colloidal solutions in water have been shown to degrade completely in 15 days in open vials [153] and that flakes of MXene can decrease in conductivity over time in air due to edge oxidation [154]. This degradation in air is a serious challenge that needs to be overcome in terms of producing long lifetime biosensors. Research focussing on the incorporation of MXenes into electrochemical sensing platforms and biosensors has been limited so far with examples mostly focussing on the detection of small molecule biomarkers, pharmaceuticals, and environmental contaminants with some of reviews previously produced [155–157]. Published work focussing on true biosensor development with MXene has focussed on the production of enzyme-based sensor platforms, predominantly for glucose utilising glucose oxidase. The high conductivities and large surface area in its unique accordion style structure offer excellent immobilisation possibilities for the enzymes in favourable microclimates for maintaining bioactivity [155].

One of the first examples of using MXene in a pure electrochemical biosensing platform for glucose was reported by Rakhi et al. [52], utilising classical amperometric detection. They prepared Ti_3_C_2_T_X_ MXene nanosheets decorated with nanocrystalline Au clusters, which served to increase the electrocatalytic performance of the overall sensing platform. They manufactured the sensor through facile drop-casting of the Au/MXene onto the surface of a polished GCE, followed by drop-casting of the enzyme/Nafion layer. This produced a glucose biosensor with a linear range between 0.1 and 18 mM with a detection limit of 5.9 μM. Ti_3_C_2_T_X_ shows metallic conductivity, excellent electrochemistry, and a high biocompatibility, making it highly suitable for the development of biosensors [157]. Lei et al. [53] took this further by developing a more oxygen-rich sensor for glucose, as common solid-liquid two phase glucose biosensors struggle to supply sufficient oxygen for superior detection levels. They utilised carbon nanotubes (CNTs) and Prussian blue (PB) along with the MXene to make a CNTs/Ti_3_C_2_T_X_/PB/CaCO_3_ film that was transferred onto a carbon fibre paper to form the electrode. Following this, the enzyme was immobilised onto the electrode along with chitosan through drop-casting (Fig. 8B). This sensor produced a working linear range in laboratory conditions of 0.2 μM–4.8 mM with a LOD of 67 nM. Research has also focussed on the best methods of MXene production [54] or the incorporation of MXene with graphene [55] for use in glucose oxidase based sensors. Ti_3_C_2_-based MXene compounds have also been reported with layered double hydroxides (LDHs) to enhance the electron transfer rate and significantly improve the conductivity of the composites towards enzyme-free glucose sensing [158]. Research into other areas of biosensing using MXene has been limited, although MXene has been used as a precursor for producing sodium titanate nanoribbons for use in a prostate specific antigen biosensor and as nanosheets in an immunosensor for carcinoembryonic antigen (CEA) [56]. In this latter work, amino functionalisation is introduced to the Ti_3_C_2_ nanosheets in order to covalently immobilise the appropriate antibodies. The composition and morphology of the MXene allow for improved biomolecule loading and faster access to the analytes, resulting in enhanced biosensor performance with a wide linear range of 0.0001–2000 ng mL^−1^ [56]. These MXenes are still at a very early stage of development and utilisation; however their wide variety of physical and chemical properties indicate that they will be a common sight among reported biosensing platforms in the future literature.

## Conclusions and outlook

Micro- and nano-dimensional materials continue to play a vital role in the development of biosensing platforms due to the beneficial properties that they bring. They help enable the sensor platforms to reach new levels of sensitivity, stability, and reliability. Trends in the field however, as with other areas, tend to bounce from material to material depending not on what will benefit detecting a specific antigen but on what is a ‘hot topic’. The use of multiple materials in single sensor platforms has become a norm and where well-reasoned and explored can help vastly improve the performance of sensors. The key is to fully explain the choice between two materials being used, explain the rationale behind the choice, and explore whether the benefit gained from the synergy between the materials outweighs the negatives of cost, production time, and complexity. The field of exploring micro- and nano-dimensional materials in biosensor platforms is vital to many of the challenges facing the world today. It will therefore need dedicated research in many areas including the development of new materials, improved incorporation of materials, synergetic combinations, and the reduced cost of implementing them commercially.
